# *Billgrantia hypersalina* sp. nov. LNSP4103-1^T^: A Halotolerant Bioplastic-Producing Bacterium from Saline Agricultural Soil

**DOI:** 10.3390/microorganisms13122683

**Published:** 2025-11-25

**Authors:** Pedro Mauricio Moran-Olvera, Joseph Guevara-Luna, Ivan Arroyo-Herrera, Violeta Larios-Serrato, César Mateo Flores-Ortiz, Luis Barbo Hernández-Portilla, Edgar López-Villegas, Paulina Estrada-de los Santos, Juan Alfredo Hernández-García, María Soledad Vásquez-Murrieta

**Affiliations:** 1 Escuela Nacional de Ciencias Biológicas, Instituto Politécnico Nacional, Prol. de Carpio y Plan de Ayala s/n, Col. Santo Tomás, Del Miguel Hidalgo, Mexico City 11340, Mexico; mauriiciio.olvera@gmail.com (P.M.M.-O.); josephguevara.28@gmail.com (J.G.-L.); ivanarroyoqbp@gmail.com (I.A.-H.); viosdatafactory@gmail.com (V.L.-S.); ivoliver@hotmail.com (E.L.-V.); pestradadelossantos@gmail.com (P.E.-d.l.S.); 2 Laboratorio de Fisiología Vegetal, Unidad de Biología, Tecnología y Prototipos (UBIPRO), Facultad de Estudios Superiores Iztacala, Universidad Nacional Autónoma de México, Av. de los Barrios No. 1, Los Reyes Iztacala, Tlalnepantla 54090, Mexico; cmflores@unam.mx; 3 Laboratorio Nacional en Salud, Facultad de Estudios Superiores Iztacala, Universidad Nacional Autónoma de México, Av. de los Barrios No. 1, Los Reyes Iztacala, Tlalnepantla 54090, Mexico; lbarbo@unam.mx

**Keywords:** Family *Halomonadaceae*, *Billgrantia*, PHA production, extreme halotolerant

## Abstract

The genus *Billgrantia* includes species with characteristics relevant to biotechnology. Several of these species can produce polyhydroxyalkanoates (PHAs), biodegradable biopolymers that play a role in adaptation to extreme conditions and have industrial applications. In this study, we describe a new species of the genus *Billgrantia* isolated from a sodium-saline soil in the community of Los Negritos, Villamar, Michoacán de Ocampo, Mexico. The strain LNSP4103-1^T^ was characterized at the genomic level using digital DNA-DNA hybridization and Average Nucleotide Identity (ANI) approaches. The difference indices between strain LNSP4103-1^T^ and the type strains (<86%) were below the threshold, supporting its classification as a novel species. The LNSP4103-1^T^ strain produced polyhydroxyalkanoates, with a maximum specific polymer yield of Y_PHA/X_ 0.71 g PHA g^−1^ biomass and a yield Y_PHA/S_ 0.56 g PHA g^−1^ glucose under conditions of 12.5% (*w*/*v*) NaCl and pH 7.5. Additionally, sucrose, mannitol, lactose and galactose were identified as substrates for PHA production. The draft genome version was deposited in the NCBI under the accession number JBMIQI000000000. The polyphasic analysis identified strain LNSP4103-1^T^ (TDS-413^T^, CAIM 1962^T^) as a new species. We proposed its taxonomic assignment as *Billgrantia hypersalina* sp. nov.

## 1. Introduction

The reclassification of bacterial species is becoming increasingly important due to advances in genomic technologies and the need for accurate taxonomic classification. Bacteria are classified in the Linnean hierarchy that ranges from domain to subspecies. Classical taxonomy employed observable phenotypic and chemotaxonomic characteristics to delimit bacterial species. However, the current taxonomy informed by comparative genomic analysis, has enabled us to reclassify species and genera. This change is crucial for understanding the evolutionary relationships and ecological functions of bacteria [1].

The *Halomonadaceae* family comprises a diverse group of halophilic and halotolerant bacteria that primarily occur in marine environments, saline soils, and saline and hypersaline lakes. This family comprises 20 genera, with the genus *Halomonas* being the most studied. Recent taxogenomic studies have revealed phylogenetic discrepancies, that have led to proposals for the reclassification of several genera within the family, including *Billgrantia*, *Bisbaumannia*, *Vreelandella*, and *Onishia*, among others [2].

The genus *Billgrantia* was first described by de la Haba et al. [2] and comprises 18 validly published names on the List of Prokaryotic Names with Standing in Nomenclature (https://lpsn.dsmz.de/, accessed on 15 September 2025); the genus *Halomonas* is a basonym, and its names remain valid. The genus consists of Gram-negative bacilli (0.3–1.1 × 0.8–6.0 µm) that are generally aerobic or facultatively anaerobic, most strains are catalase and oxidase positive, with colonies exhibiting brown, yellow, white, beige, and pinkish-white pigmentation. The bacteria are mesophilic, capable of growing at 0–26% (*w*/*v*) NaCl (optimal range of 1–15%) and 4–55 °C (with an optimal range of 25–42 °C). Members of the genus *Billgrantia* exhibit metabolic versatility related to the various environments from which they were isolated, such as deep-sea sediments, ocean hydrothermal vents, seawater samples from caves, lakes, soil, saline soil, liquid collected from *Populus euphratica* stems, oil-contaminated saline soil, alkaline lakes, hypersaline lakes and salted skins [2].

Among the main biotechnological applications of bacteria are the production of enzymes, including cellulases, lipases and proteases, the production of biosurfactants and extracellular polysaccharides, compatible solutes such as ectoine used as a protective agent in medical applications, as and the production of bioplastics including polyhydroxyalkanoates (PHAs) [3].

Polyhydroxyalkanoates are a class of biopolymers produced by various microorganisms, including bacteria, archaea, and algae; they are biodegradable and decompose into water and carbon dioxide under microbial action. This makes them an environmentally friendly alternative to non-biodegradable plastics. Certain PHAs meet marine biodegradability standards, meaning they have short persistence in marine environments [4], thereby reducing plastic pollution and consequently their environmental impact.

The large-scale production of polyhydroxyalkanoates (PHAs) faces significant technological and economic constraints that hinder their market competitiveness. Substrate cost remains one of the main bottlenecks, as it often represents more than 40–50% of the overall production cost. Additionally, low productivity, challenges in process scalability, and expensive downstream recovery steps substantially increase operational costs. The variation in PHA composition and physicochemical properties depending on the microbial strain and feedstock also limits standardization for industrial applications. Consequently, the overall production cost of PHAs (US$4000–15,000 per ton) remains considerably higher than that of conventional petrochemical plastics (~US$1250 per ton), delaying their widespread adoption until improvements in production costs are achieved [5].

PHA biopolymers are synthesized by the microorganisms as carbon and energy reserves under nutrient-limited conditions and accumulate in the form of granules within cells know as ‘carbonosomes’. After the reclassification of the *Halomonadaceae* family, the remaining genera that produced PHAs were *Billgrantia*, *Vreelandella* (formerly *Halomonas*) and *Cobetia* [2,3]. The subsequent section presents data compiled from various members of the Halomonadaceae family capable of PHA production (Table 1).

The objective of this study was to perform a polyphasic analysis of strain LNSP4103-1^T^ isolated from saline-sodic soil in Los Negritos, Michoacán de Ocampo, a new species of *Billgrantia*, a PHA producer.

## 2. Materials and Methods

### 2.1. Isolation and Reactivation of Microbial Resources

The strain was isolated from soil samples collected in Los Negritos, in the municipality of Villamar in Michoacán de Ocampo (20°03.780′ N 102°36.819′ W) [12]. The strain was reactivated by inoculating 10 μL of the vial provided in 10% SP medium (g L^−1^): NaCl 98, KCl 2.0, MgSO_4_•7H_2_O 1.0, CaCl_2_•2H_2_O 0.36, NaHCO_3_ 0.06, NaBr 0.23, peptone 5.0, yeast extract 10.0, glucose 1.0 at pH 7.0 [13] by cross-streaking and incubating at 28–30 °C for 48 h.

### 2.2. Analysis of the 16S rRNA Gene Sequence

The bacteria were obtained from a culture grown in Tryptic Soy Broth (TSB) with agitation (150 rpm) for 24 h. DNA was extracted from the culture using the phenol-chloroform method [14]. The 16S rRNA gene was amplified using the universal primers 27F (5′-AGAGTTTGATCCTGGCTCAG-3′) and 1492R (5′-GGTTACCTTGTTACGACTT-3′). A 1458 bp amplification product was obtained and verified by electrophoresis on 1% agarose gels. The PCR products were sent to Macrogen (www.macrogen.com, Seoul, Republic of Korea). The 16S rRNA gene sequence was analyzed and manually edited using Seaview v.5. [15] and compared to databases using the EzBioCloud platform (www.ezbiocloud.net/). The sequences that showed the highest percentage of identity (>80%) were downloaded to perform phylogenetic reconstruction. The MUSCLE tool from the Ugene software [16] was used for sequence alignment. Phylogenetic reconstruction was performed using the Maximum Likelihood method with IQ-TREE2 version 2.1.2 software [17]. The nucleotide substitution model that best fit each of the data sets was automatically estimated, and the robustness of the trees was assessed using 1000 bootstrap replicates. A similarity matrix was estimated using the optimal dynamic alignment method of Myers and Miller, as implemented in the MatGat version 2.1 software [18].

### 2.3. Genomic Analysis

Extraction of genomic DNA was performed using a Quick-DNA Fungal/Bacterial Miniprep Kit (Zymo Research, Irvine, CA, USA) following the manufacturer’s instructions. The purified DNA was sent to Novogene (San Diego, CA, USA) for sequencing with 150 bp paired-end reads using the Illumina Novaseq platform (Illumina, San Diego, CA, USA). The quality of the raw sequences was assessed using FastQC v.0.12.0 [19]. Trimming and adapter removal of the raw data were performed using Trimmomatic v.0.39 [20], with a Phred quality score of 33. The sample was assembled de novo using an optimized de Bruijn graph method and automatic K value adjustment using the SPAdes v.3.14.1 software [21]. The search for mobile elements, plasmids and viral genomes in strain LNSP4103-1^T^ was performed using the geNomad software v.0.4.0 with default parameters on the NMDC EDGE web platform (https://nmdc-edge.org/, accessed on 5 March 2025) [22]. CheckM2 v.1.0.2 [23] was used to assess the quality and percentage contamination of the assembled genome. The annotation of the *Billgrantia* strain was performed with Prokka v.1.16.1 [24]. GenoVi v.0.4.3 [25] was used to create a circular map of the LNSP4103-1^T^ strain, allowing for a visual assessment of the genome’s characteristics. Functional annotation was performed with the COG software v.1.2.3 (https://github.com/robotoD/GenoVi).

The overall genomic relationship indices (OGRIs) calculated were the average nucleotide identity index (ANI) by pyANI v.0.2.9 (https://github.com/widdowquinn/pyani, accessed on 6 March 2025) using ANIb (based on the use of BLAST v.2.16.0) and ANIm (based on the use of the MUMmer software v.4.0+), the tetranucleotide signature frequency correlation coefficient (TETRA) [26], FastANI (based on Mashmap and MinHash) [27], and the average amino acid identity index (AAI) using the EzAAI program (based on the MMSeqs2 software v.2-15. https://github.com/endixk/ezaai, accessed on 25 June 2025) [28]. The threshold criteria for defining a new species were ≤95% identity for ANIb, ANIm, and FastANI; 0.999 for TETRA, and <95% AAI [29,30]. Digital DNA-DNA hybridization (dDDH) was calculated in silico using the Genome-to-Genome Distance Calculator (GGDC 3.0) with the BLAST method (https://tygs.dsmz.de/, accessed on 14 September 2023). The results were based on the recommended formula 2 (identities/length of high-score pair), which is independent of genome length and recommended for incomplete genomes [31]. Phylogenomic reconstruction was performed using two methods. The first employed OrthoFinder v.3.0.1b1 [32] based on inferring orthogroups, as well as the complete set of genetic trees for all orthogroups. The second method utilized DIAMOND v. 2.1.11 [33] to perform identity searches within the orthologous proteins obtained, considering only proteins with a similaraty percentage of greater than 90%. *Chromohalobacter marismortui* DSM 6770^T^ and *Salinicola socius* DSM 19940^T^ were used as outgroups. The bootstrap calculation was performed using the alignment obtained with OrthoFinder in the IQ-TREE2 software. The reconstructed phylogeny was visualized in iTOL v.7 (https://itol.embl.de/, accessed on 23 September 2025) [34].

The biosynthetic pathway of PHAs was reconstructed using metabolic maps obtained from the BlastKOALA platform (https://www.kegg.jp/blastkoala/, accessed on 27 September 2025) and the pathway mapper of the KEGG platform (https://www.kegg.jp/, accessed on 27 September 2025), in which information obtained from the annotation of the strains was used. The genes and enzymes directly involved in the biosynthesis of metabolites related to salt stress, ‘*salt-out*’ and ‘*salt-in*’ strategies, were also identified [35].

### 2.4. Phenotypic Analysis

Morphological and physiological characterization was carried out by culturing strain LNSP4103-1^T^ in Luria–Bertani (LB) medium, trypticase soy agar (TSA), and SP 10% for morphological description. The microscopic observations were obtained after negative staining using a JEOL transmission electron microscope (Model JSM1010, JEOL, Tokyo, Japan) at an acceleration voltage of 60,000 V. LB medium was then used to evaluate bacterial growth at different temperatures (10, 20, 28, 30, 35, 37, 40, 45, and 50 °C), with growth observed at 24 and 48 h. The effect of pH was evaluated in 180 µL of LB broth using the following buffers: 0.1 M glycine with 0.1 M HCl (pH 1–2), 0.1 M citric acid with 0.2 M Na_2_HPO_4_ (pH 3–7), 0.0667 M KH_2_PO_4_ with 0.0667 M NaH_2_PO_4_ (pH 8), and 0.1 M glycine with 0.1 M NaOH (pH 9–13). Uninoculated medium was used as a control. The pH was measured and adjusted after sterilization of the medium if necessary. A 20 μL aliquot of the cell suspension with an OD_600_ of approximately 0.900 was inoculated into a 96-well microplate in triplicate and incubated with shaking at 150 rpm for four days. Growth was estimated spectrophotometrically at OD_600_ using a Multiskan FC microplate photometer (Thermo Fisher Scientific, Waltham, MA, USA). NaCl tolerance was tested using 180 µL of LB broth by modifying the NaCl concentration (*w*/*v*) from 0% to 20%, in increments of 1%, and at 25% and 30%. Different carbon sources were used with 180 µL of mineral salt broth (MSM) composed of (g L^−1^): 1.5 K_2_HPO_4_, 0.5 KH_2_PO_4_, 1.0 NH_4_NO_3_, and 30 NaCl, with the pH adjusted to 7.0–7.2 and supplemented with 0.5% carbohydrates (L-arabinose, D-fructose, D-galactose, D-glucose, glycerol, α-lactose, malic acid, D-maltose, D-mannose, propionic acid, D-ribose, sodium acetate, sodium citrate, sodium gluconate, sodium L-lactate, D-sorbitol, succinic acid, D-sucrose, and D-xylose) and 0.1% amino acids (L-alanine, L-arginine, L-asparagine, L-glutamate, glycine, L-isoleucine, L-lysine, L-ornithine, L-proline). A 20 μL aliquot of cell suspension at a OD600 of approximately 0.900 was inoculated into a 96-well microplate in triplicate and incubated with shaking at 150 rpm for six days [36]. Antibiotic susceptibility was tested using the Kirby-Bauer method by CLSI (2024) (https//clsi.org/). Sensidiscs for antibiograms contained the following antibiotics: amikacin (30 µg), ampicillin (30 µg), carbenicillin (100 µg), cephalothin (30 µg), cefotaxime (30 µg), ciprofloxacin (5 µg), chloramphenicol (30 µg), gentamicin (10 µg), netilmicin (30 µg), nitrofurantoin (300 µg), norfloxacin (10 µg), and trimethoprim with sulfamethoxazole (25 µg). The plates were checked after 24 h. Catalase activity was tested by placing an isolated colony from a 24-h culture on a slide and then adding one drop of 3% hydrogen peroxide. Oxidase activity was verified using Bactident^®^ Oxidase strips (MERCK, Darmstadt, Germany) [3]. The following biochemical tests were performed according to Ventosa et al. [3], using a series of NaCl concentrations (0–10%): Methyl red + Voges Proskauer, NO_3_ reduction, phenylalanine deaminase, lysine, iron, agar (LIA), urea broth (Christensen), motility, indole, ornithine (MIO), oxidation–fermentation (Hugh-Leifson), and Kligler iron agar.

The effects of environmental factors on the production of PHA were evaluated using 9 mL of modified accumulation medium (MAM) (g L^−1^): 0.5 yeast extract, 20 glucose, 240 NaCl, 30 MgCl_2_•6H_2_O, 35 MgSO_4_•6H_2_O, and 7 KCl [37] at 5% salinity. The initial pH was adjusted to 5, 6, 7, 8, 9, and 10 using 2 M KOH and 0.1 N HCl, with pH 7.5 as the control. A 1 mL aliquot of cell suspension at an OD_600_ of approximately 0.900 was inoculated into vials in triplicate and incubated with shaking at 150 rpm. Biomass samples were collected at 0, 12, 24, 48, 72, and 96 h, and the biomass and amount of PHA were quantified following the methodology reported by Sav et al. [38]. Starting from the dry biomass, 2 mL of pure chloroform was added and stirred at 200 rpm for 48 h at 37 °C. The chloroform was recovered by filtering out the biomass, and 8 mL of ethanol was added to precipitate the PHA. The biomass and PHA produced were quantified by dry weight. Once the optimal pH and time of collection had been determined, the salinity was evaluated (0, 5, 7.5, 10, 12.5% (*w*/*v*)), considering 5% as the control; the preparation of the inoculum and the quantification methodology were carried out as specified for the evaluation of pH, but with the samples collected at 0, 24, 48 and 72 h. The evaluation of various carbon sources (galactose, lactose, mannitol, and sucrose) was conducted following the determination of the optimal NaCl concentration, with glucose serving as the control. The preparation of the inoculum and the quantification methodology were as previously described.

### 2.5. Chemotaxonomic Analysis

Fatty acids were determined using a Model 6850 gas chromatograph coupled to a Model 5975C mass spectrometer, both from Agilent Technologies (Santa Clara, CA, USA). A 30-m-long HP-5MS capillary column with a diameter of 0.25 µm and a film thickness of 0.25 µm was used, with an initial oven temperature of 150 °C. The initial temperature was maintained for two minutes, followed by a heating ramp of 5 °C min^−1^ to 200 °C and a second ramp of 3 °C min^−1^ to 240 °C, with a total run time of 25.33 min. The injector temperature was 250 °C, and splitless injection mode was employed with a purge time of 0.3 min. The carrier gas used was ultra-high-purity helium with a flow rate of 1 mL/min. The mass spectrometer acquisition parameters were positive ions by electron impact at 70 eV, with an ionization source temperature of 230 °C and a quadrupole temperature of 150 °C. The mass range was 35 to 400 m z^−1^. The injection volume of the samples was 2 µL [39].

The analysis of the predominant ubiquinone ‘in silico’ was performed by phylogenetic reconstruction of the amino acid sequences corresponding to the biosynthetic pathway of the isoprene units that determine the chain length of the predominant ubiquinone (ispB, ispA) [40]. The analyses were performed using Ugene v.51.0, with the MUSCLE alignment tool. The proteins used from ispB for the eight isoprene units responsible for Q-8 were taken from *Escherichia coli* K-12^T^; for the nine isoprene units responsible for Q-9, these were from *Halomonas elongata* DSM 2581^T^, *Halomonas ventosae* CECT 5797^T^, *Kushneria konosiri* X49^T^, *Billgrantia desiderata* CIP 105505^T^, and *Billgrantia ethanolica* KCTC 72090^T^. The ispA proteins for the 10 isoprene units responsible for Q-10 were taken from *Cereibacter sphaeroides* 2.4.1^T^. The phylogenetic tree was reconstructed using IQTREE v.2.0.3. (https://github.com/iqtree/iqtree2, accessed on 14 March 2025) and visualized in iTOL version 7.

Polar lipids were extracted using the method described by Bligh and Dyer [41]. The polar lipids were identified by two-dimensional thin-layer chromatography [42] using sulfuric acid–ethanol (30%, *v*/*v*). Phosphoric acid, ninhydrin, and the periodate-Schiff technique were used for the detection of total lipids, phospholipids, aminolipids, and glycolipids, respectively [4,42].

### 2.6. Statistical Analysis

The normality of the data was assessed using the Shapiro–Wilk test, while the homogeneity of variance was checked using Levene’s test. The data on polyhydroxyalkanoate production were subjected to one-way analysis of variance (ANOVA) to determine whether there were significant differences between production kinetics using the R software package (www.r-project.org). The reported values correspond to the mean of three independent replicates for each sampling time. A *p*-value < 0.05 was considered significant.

## 3. Results

### 3.1. Analysis of the 16S rRNA Gene Sequence

According to the 16S rRNA gene sequence comparison, the closest species to strain LNSP4103-1^T^ were *Billgrantia sulfidoxydans* CYN-1-2^T^ (99.27%), *Billgrantia tianxiuensis* BC-M4-5^T^ (99.12%), and *Billgrantia ethanolica* CYT3-1-1^T^ (98.90%). Strain LNSP4103-1^T^ showed high similarity to *B. sulfidoxydans* CYN-1-2^T^ (92.9%, Table S1), *B. tianxiuensis* BC-M4-5^T^ (92.8%), and *B. ethanolica* CYT3-1-1^T^ (92.6%).

**Figure 1:**
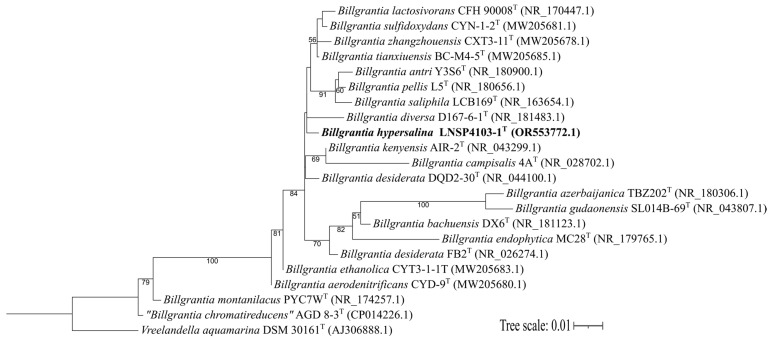
Phylogenetic reconstruction by the maximum likelihood method based on 16S rRNA gene sequences showing the relationships between 20 type strains of the genus *Billgrantia* and the study strain. Likelihoods were calculated with the HKY + F + I + G4 model. Nodes show bootstrap percentages ≥ 50% (1000 replicates). The tree scale is 0.01 substitutions per nucleotide. *Vreelandella aquamarina* DSM 30161^T^ was used as the outgroup.

A partial representation of the maximum likelihood phylogenetic reconstruction shows the distribution of strain LNSP4103-1^T^ and its relationship to the other strains (Figure 1). Analyses of the 16S rRNA gene sequences identified strain LNSP4103-1^T^ as a new species of the genus *Billgrantia*.

### 3.2. Genomic Analysis

The draft genome of strain LNSP4103-1^T^ included 21 contigs and had a size of 4,180,479 bp, with a G + C content of 63.75 mol%. Whole genome sequencing (WGS) identified 3832 protein-coding genes and 59 RNA genes. The strain LNSP4103-1^T^ was compared using different OGRIs, showing ANI values below the cut-off limits (ANIb: 79.05–84.70%; ANIm: 84.52–86.81%), AAI: 79.2–87.8%, and dDDH: 25.2–28.3%. In comparison to related species of the genus *Billgrantia*, *B. lactosivorans* KCTC 52281^T^ obtained the highest scores among the calculated indices, representing the closest species. Therefore, following the cut-off limits established for the description of a new species (ANI < 96%, AAI < 95%, and dDDH < 70%), the strain was considered a new species belonging to the genus *Billgrantia* (Table 2).

**Figure 2:**
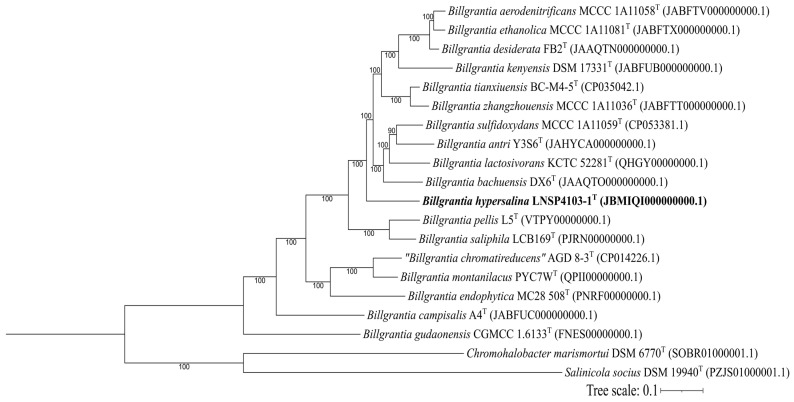
Phylogenomic reconstruction by the maximum likelihood method using OrthoFinder. The reconstruction assigned 5818 orthogroups comprising 78,210 shared genes. Likelihoods were calculated with the LG + F + I + R4 model. Bootstrap percentages ≥ 50% (based on 1000 replicates) are shown at the nodes. The tree scale is 0.1 substitutions per amino acid. *Chromohalobacter marismortui* DSM 6770^T^ and *Salinicola socius* DSM19940^T^ were used as outgroups.

Orthologous gene analysis was performed using OrthoFinder (Figure 2), resulting in a total of 78,210 single-copy orthologous genes distributed across 5818 orthogroups shared among members of the genus *Billgrantia*. However, when employing the cut-off, a total of 300 genes with percentages of identity greater than 90% were identified between strain LNSP4103-1^T^ and the other members of the genus. The genes identified were primarily related to central metabolism.

**Figure 3:**
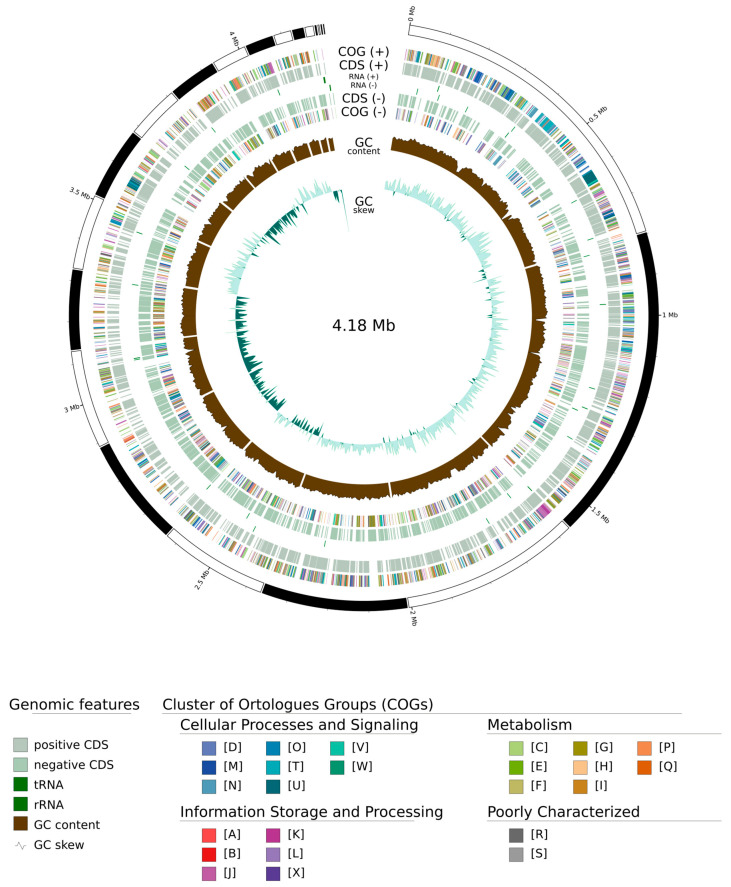
Illustration of the distribution of the annotated *Billgrantia hypersalina* LNSP4103-1^T^ genome. The elements shown in pale green correspond to the CDS, and the tRNA and rRNAs are shown in deep green. The barcode set with different colors represents COG annotations. The graph was visualized with GenoVi.

In addition, a circular map was constructed with the following characteristics: estimated sequence length, positive and negative chain coding genes, tRNA, rRNA, GC content, and COG annotation distributed in different colors (Figure 3).

The metabolic pathway for polyhydroxybutyrate synthesis was reconstructed using BlastKOALA (Supplementary Material Figure S1, Table S2). The metabolic pathway for PHA synthesis begins with glycolysis to produce pyruvate. Then, the enzyme pyruvate dehydrogenase catalyzes the reaction to obtain 2-hydroxyethyl-ThPP, and the same enzyme acts to produce S-Acetyl-dihydrolipoamide-E. At this point, the dihydrolipoil lysine acetyltransferase enzyme produces acetyl-CoA. Acetyl-CoA is catalysed by the acetyl-CoA acetyltransferase enzyme to produce acetoacyl-CoA. From here, the LNSP4103-1^T^ strain can follow two routes for the formation of PHB. In the first step, the enzyme 3-hydroxyacyl-CoA dehydrogenase participates in the production of (S)-3-hydroxybutanoyl-CoA, after which 3-hydroxybutyryl-CoA epimerase rotates the molecule to obtain (R)-3-hydroxybutanoyl-CoA. In the second pathway, the enzyme acetoacetyl-CoA reductase allows the direct production of (R)-3-hydroxybutanoyl-CoA. In both cases, the polyhydroxyalkanoate synthase subunit is responsible for producing poly-β-hydroxybutyrate.

Thirty-one genes related to the ‘*salt-out*’ strategy were identified (Table S3), including genes involved in the metabolism of betaine-choline (15 genes), ectoine (nine genes), and glutamate-proline (seven genes). There were 27 genes related to the ‘*salt-in*’ strategy (Table S4), including potassium homeostasis (six genes), osmoregulation (five genes), and Na^+^ and K^+^ ion efflux pumps (16 genes).

### 3.3. Phenotypic Analysis

**Figure 4:**
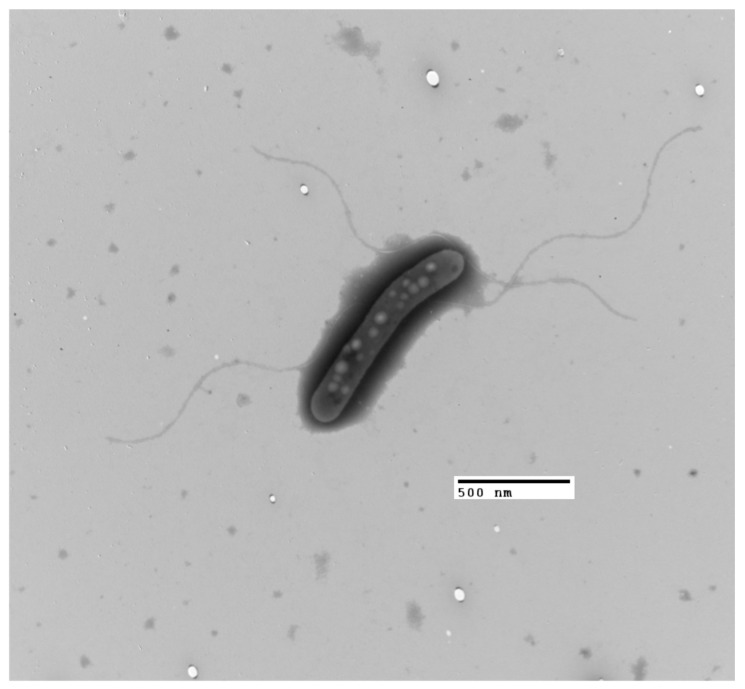
Microscopic examination of the morphology of the strain LNSP4103-1^T^ processed using the negative staining technique and observed under a transmission electron microscope (TEM) at 10,000×.

The LNSP4103-1^T^ strain is a Gram-negative, short bacillus-shaped bacterium, 0.51 × 3.87 µm (Figure 4). The bacterium is strictly aerobic and halotolerant. The colonies are circular, flat, moist in appearance, mucoid in consistency, and appear yellow in color after 72 h when grown on TSA with 10% NaCl (Figure S2).

Growth was observed at temperatures of 4–37 °C (optimum range = 28–30 °C), pH 7–12 (optimum pH range = 7–8), and NaCl 0–30% (optimum 0–12%). Oxidase and catalase were positive. The bacteria produce polyhydroxyalkanoates (PHAs), DNases, esterases, and lipases at 15% (*w*/*v*) NaCl, but do not produce 3-indoleacetic acid. The following biochemical tests were positive in the presence of NaCl (2.5–10%): nitrate reduction, H_2_S production, lysine decarboxylase, ornithine decarboxylase, and phenylalanine deaminase, and negative for Vogues-Proskauer, methyl red, and urease. The bacteria were susceptible to all antibiotics tested. The LNSP4103-1^T^ strain could assimilate D-glucose, D-mannose, D-fructose, propionic acid, sodium acetate, sodium gluconate, mannitol, L-lactate, D-sucrose, sodium citrate, L-isoleucine, L-lysine, and L-arginine, but not glycerol, D-xylose, succinic acid, D-ribose, malic acid, D-maltose, starch, L-arabinose, D-sorbitol, α-lactose, D-galactose, L-proline, L-asparagine, glycine, L-ornithine, L-alanine, or L-glutamate (Table 3).

The LNSP4103-1*^T^* strain produced polyhydroxyalkanoates with a maximum yield of Y_PHA/X_ 0.71 g PHA g^−1^ biomass at 24 h, under conditions of 12.5% (*w*/*v*) NaCl and pH 7.5, using glucose as the substrate. In contrast, the maximum yields obtained with alternative carbon sources were all below 50% at 48 h: sucrose yielded 0.28 g PHA g^−1^ biomass, mannitol yielded 0.26 g PHA g^−1^ biomass, lactose yielded 0.18 g PHA g^−1^ biomass, and galactose yielded 0.13 g PHA g^−1^ biomass.

### 3.4. Chemotaxonomic Analysis

The predominant fatty acids identified for strain LNSP4103-1^T^ were as follows: C_16:0_ (42.775%), C_18:0_ (33.935%), C_18:1_ w9c (10.351%), and, to a lesser extent, C_18:1_ w9t (9.138%), C_14:0_ (1.564%), C_16:1_ w9c (1.212%), and C_18:2_ w9,12 (1.024%). The major polar lipids detected were diphosphatidylglycerol (DPG), phosphatidylethanolamine (PE), and phosphatidylglycerol (PG) (Figure S3). The phylogenetic reconstruction of the ispB (Q-8 and Q-9) and ispA (Q-10) proteins responsible for the isoprene units for the predominant ubiquinones incorporated strains that were closely related to the LNSP4103-1^T^ strain, as well as other members of the *Halomonadaceae* family (Figure S4). By adding protein sequences from the predominant ubiquinone of *E. coli* K-12^T^ (Q-8) and *C. sphaeroides* 2.4.1^T^ (Q-10), it was possible to differentiate between the clades of ubiquinones 8 and ubiquinones 9 and 10.

## 4. Discussion

### 4.1. Proposal of Billgrantia hypersalina sp. nov.

Analyses of the 16S rRNA gene sequences identified strain LNSP4103-1^T^ as a new species of the genus *Billgrantia*. The G + C (mol%) content was consistent with that reported for the genus *Billgrantia* (62.1–67.5 mol%) [2]. Therefore, following the cut-off limits established for the description of a new species (ANI < 96%, AAI < 95%, and dDDH < 70%), we determined that the strain was a new species belonging to the genus *Billgrantia*. Analysis of the OGRIs in conjunction with the phylogeny of the 16S gene and the phylogenomic reconstruction demonstrated that strain LNSP4103-1^T^ represents a new species in the genus *Billgrantia*.

The major polar lipids for the genus *Billgrantia* [2] are diphosphatidylglycerol, phosphatidylglycerol, and phosphatidylethanolamine. Our comparative analysis demonstrated that the polar lipid profile of the studied strain aligned closely with that reported for the genus *Billgrantia*, further substantiating its taxonomic placement within this genus.

The variation within species of the genus *Billgrantia* may be related to the isolation site or the culture medium used for fatty acid identification, as the latter varies according to the nutritional composition of the culture medium [43].

Previous studies have established that the predominant ubiquinones are highly conserved protein structures primarily related to the environment in which the bacteria develop, with ubiquinone-9 being the most notable exception, particularly in relation to the stress generated by saline environments [40]. However, there are cases in which ubiquinone-10 has been established as the predominant for some bacteria belonging to the Alphaproteobacteria class, as well as for eukaryotic cells [44].

### 4.2. Description of Billgrantia hypersalina sp. nov.

*Billgrantia hypersalina* (hy.per.sa.li’na. Gr. prep. *hyper*, above; N.L. masc. adj. *salinus*; N.L. fem. adj. *hypersalina*).

The cells are Gram-negative, short bacillus-shaped, strictly aerobic, and halotolerant. Colonies on TSA are circular, flat, moist in appearance, mucoid in consistency, and yellow in color. The bacteria grow between 10 °C and 30 °C, at a pH of 7–12, in presence of and 0–30% (*w*/*v*) NaCl. Oxidase and catalase are positive. The bacteria produce polyhydroxyalkanoates (PHA). *B. hypersalina* reduces nitrates, produces H_2_S, has oxidative metabolism, and does not produce indole. The most abundant fatty acids are C_16:0_, C_18:0_, and C_18:1_ w9c.

The G + C content of the strain is 63.75 mol%. The strain was isolated from saline-sodic soil from the community of Los Negritos, Michoacán de Ocampo, Mexico, and deposited at the ATCC (TDS-413^T^) and CAIM (CAIM 1962^T^).

### 4.3. Metabolic Pathway, Production of Polyhydroxyalkanoates, and Osmoadaptation Mechanisms

The reconstructed metabolic pathway was compared with one of the pathways published by Choi et al. [45] which involves the production of polyhydroxybutyrate from carbohydrates. The final PHA yield of strain LNSP4103-1^T^ was compared to the production of 0.82 g of PHA g^−1^ biomass at 48 h obtained by Tan et al. [46] for *Halomonas bluephagenensis* and 0.47 g PHA g^−1^ biomass at 72 h reported by Kucera et al. [6] for *Halomonas halophila* using glucose as the substrate.

The persistence of intracellular polyhydroxyalkanoate (PHA) granules following transfer to carbon-deficient media is extensively documented. Notably, investigations involving *Halomonas bluephagenesis* [46] and *Cupriavidus necator* [47] have demonstrated that these reserve polymers remain largely unaltered during periods of nutrient deprivation or incubation in non-inductive conditions, without an appreciable reduction in PHA content. Therefore, the presence of the polymer in cultures supplemented with galactose or lactose reflects the metabolic inheritance from the inoculum rather than de novo biosynthetic induction by these alternative carbon sources. This phenomenon is also evident among halophilic members of the *Halomonadaceae* family, where the accumulation of PHA predominantly occurs during cultivation with glucose or fatty acids, and the polymer persists intracellularly even after cells are transferred to non-permissive environments [48]. In such instances, the absence of new monomer incorporation and the constant cell mass confirm that the observed PHA originates from previously accumulated reserves. Collectively, these findings indicate that strain LNSP4103-1^T^ does not utilize galactose or lactose for growth or PHA biosynthesis; instead, the detected polymer in these experimental conditions is likely residual from prior glucose-based cultivation. This conclusion aligns with observations reported for other halophilic PHA-producing organisms [46,48].

The osmoadaptation mechanisms are particularly valuable for industrial biotechnology due to halotolerant strains, which demonstrate high salt tolerance with active biosynthetic capacity. They can operate under non-sterile, high-salinity conditions or utilize saline water sources, reducing freshwater demand and sterilization costs [49].

## 5. Conclusions

In this study, *Billgrantia hypersalina* LNSP4103-1^T^ is described as a new species isolated from saline-sodic soil. Polyphasic analysis indicated that strain LNSP4103-1^T^ exhibited a sufficient degree of difference to warrant its designation as a new species. In addition, the production of polyhydroxyalkanoates and extracellular polymeric substances was confirmed.

## Figures and Tables

**Table 1:** Production of PHAs on different members of *Halomonadaceae* family.

Substrate	Organism	Type of PHA	Concentration	Reference
Cheese whey hydrolysate, corn stover.	*Halomonas halophila*	P(3HB)	82% PHB (*w*/*w* to CDW).	[6]
Glucose	*Halomonas elongata*	P(3HB)	21.2 g L^−1^ (P(3HB), 53.0% (*w*/*w* CDW).	[7]
Glucose	*Halomonas* sp. SF2003	P(3HB)/P(3HB-co-3HV)	~2.25 g L^−1^	[8]
Cheese whey mother liquor	*Halomonas alkaliantarctica*	P(3HB)/P(3HB-co-3HV)	~20.1% CDM at 72 h	[9]
Waste frying oil	*Halomonas hydrothermalis*	P(3HB) y P(3HB-co-3HV)	0.42 g/L, 50.15 mol% 3 HV fraction	[10]
Acetate, glycerol, glucose	*Cobetia marina*	PHB/PHBV	~2.5 g PHB/L, 61% with acetate.	[11]

**Table 2:** Overall Genome Relatedness Index values of the *B. hypersalina* LNSP4103-1^T^ compared to closely related species.

Strain	dDDH ^a^ (%)	ANIb ^b^ (%)	ANIm ^c^ (%)	FastANI ^d^ (%)	TETRA ^e^	AAI ^f^ (%)
*B. lactosivorans* KCTC 52281^T^	28.3	84.60	86.79	85.87	0.9630	87.43
*B. sulfidoxydans* MCCC 1A11059^T^	28.2	84.70	86.81	85.77	0.9734	87.76
*B. bachuensis* DX6^T^	27.4	84.06	86.43	84.95	0.9957	87.83
*B. tianxiuensis* BC-M4-5^T^	27.3	83.80	86.25	84.80	0.9929	87.45
*B. desiderata* FB2^T^	26.8	83.50	86.27	84.68	0.9745	86.32
*B. antri* Y3S6^T^	26.8	83.54	86.22	84.69	0.9943	87.30

^a^: Digital DNA-DNA hybridization. ^b^: Average nucleotide identity (blast). ^c^: Average nucleotide identity (MUMmer). ^d^: Average nucleotide identity (Mashmap and MinHash). ^e^: Tetranucleotide signature frequency correlation coefficient. ^f^: Average aminoacidic identity.

**Table 3:** Phenotypic and biochemical characteristics of strain LNSP4103-1^T^ that differentiate the bacteria from the type strains of closely related species of the genus *Billgrantia*.

Substrate	LNSP4103-1^T^	KCTC 52281^T^	MCCC 1A11059^T^	FB2^T^
Mobility	−	+	+	+
Pigmentation	Yellow	Yellow	Yellow	White
EPS	+	+	+	NA
PHA	+	+	+	+
NaCl range (optimal, %, p/v)	0–30.0 (0–12.0)	1.0–12.0 (4.0–10.0)	0–18.0 (2.0–6.0)	0.0–18.0 (8.0–9.0)
pH range (optimal)	7.0–12.0 (7.0–8.0)	6.0–9.0 (7.0–8.0)	6.0–10.0 (7.0–8.0)	7.0–11.0 (9.0–9.5)
Oxidase	+	+	+	+
Reduction of NO_3_	+	+	+	+
Production of indole	−	−	−	−
Production of H_2_S	+	−	−	NA
Methyl red	−	−	w	NA
Voges–Proskauer	−	−	w	−
Urease	−	−	−	NA
Lysine decarboxylase	+	−	−	NA
Ornithine decarboxylase	+	−	−	NA
Phenylalanine deaminase	+	−	NA	+
**Use of carbohydrates**				
D-glucose	+	+	+	+
Glycerol	−	+	+	+
D-xylose	−	NA	NA	+
Succinic acid	−	NA	+	+
D-ribose	−	NA	NA	+
D-mannose	+	+	−	+
Malic acid	−	NA	+	NA
D-maltose	−	+	+	+
Starch	−	−	−	−
D-fructose	+	+	NA	NA
Mannitol	+	+	+	NA
Propionic acid	+	NA	+	NA
Acetate	+	NA	+	+
Sodium gluconate	+	NA	+	+
L-lactate	+	NA	+	+
L-arabinose	−	+	+	+
D-sorbitol	−	+	NA	NA
D-sucrose	+	+	+	+
α-lactose	−	+	NA	NA
Citrate	+	−	+	+
D-galactose	−	+	NA	+
%G + C	63.75	66.7	66	66
Environment	Saline-sodic soil	Salt lake	Coastal surface sediments	Municipal sewerage

**NA**: No data available; **w**: weak; **LNSP4103-1^T^**: *B. hypersalina*; **KCTC 52281^T^**: *B. lactosivorans*; **MCCC 1A11059^T^**: *B. sulfidoxydans*; **FB2^T^**: *B. desiderata* [3].

## Data Availability

The original contributions presented in this study are included in the article and Supplementary Material. Further inquiries can be directed to the corresponding authors. The corresponding GenBank/EMBL/DDBJ accession numbers for the 16S rRNA gene, and the draft whole genome sequences and sequence read archive are OR553772.1, JBMIQI000000000 and SRR32885079, respectively.

## Supplementary Materials

The following supporting information can be downloaded at https://www.mdpi.com/article/10.3390/microorganisms13122683/s1, Figure S1: Metabolic pathway for PHA production obtained from KEGG mapper, green rectangles indicate that strain LNSP4103-1T possesses the enzyme; Figure S2: Colony morphology of the *Billgrantia hypersalina* LNSP4103-1T in TSA after 72 h incubation at 30 °C; Figure S3: Polar lipid profile of strain LNSP4103-1T; major polar lipids detected were diphosphatidylglycerol (DPG), phosphatidylethanolamine (PE), and phosphatidylglycerol (PG); Figure S4: Phylogenetic reconstruction of the protein octaprenyl diphosphate synthase (ispB); Table S1: 16S rRNA gene similarity matrix for members of the genus *Billgrantia*; Table S2: Enzymes are involved in PHA metabolism; Table S3: Genes involved in “*salt-out*” strategies or compatible solute production in strain LNSP4103-1T; Table S4: Genes involved in the “*salt-in*” strategies of strain LNSP4103-1T.

## Abbreviations

The following abbreviations are used in this manuscript:

PHA	Polyhydroxyalkanoates
DPG	Diphosphatidylglycerol
PE	Phosphatidylethanolamine
PG	Phosphatidylglycerol
ANI	Average Nucleotide Identity
TSB	Tryptic Soy Broth
PCR	Polymerase Chain Reaction
AAI	Average Aminoacidic Identity
GGDC	Genome-to-Genome Distance Calculator
BLAST	Basic Local Alignment Search Tool
KEGG	Kyoto Encyclopedia of Genes and Genomes
LB	Luria–Bertani
TSA	Tryptic Soy Agar
OD	Optical Density
MSM	Mineral Salt Broth
CLSI	Clinical & Laboratory Standards Institute
MAM	Modified Accumulation Medium
dDDH	Digital DNA-DNA Hybridization
TETRA	Tetranucleotide Signature Frequency Correlation Coefficient
GC	Guanine–Cytosine
COG	Clusters of Orthologous Genes
CDS	Coding DNA Sequence
ATCC	American Type Culture Collection
CAIM	Collection of Aquatic Important Microorganisms
