# Supplementary material for: Billgrantia hypersalina sp. nov. LNSP4103-1T: A Halotolerant Bioplastic-Producing Bacterium from Saline Agricultural Soil
Source: Microorganisms. 2025 Nov 25;13(12):2683. doi: 10.3390/microorganisms13122683 (PMC12735797; doi:10.3390/microorganisms13122683)
Supplement: Supplementary file 1 [file microorganisms-13-02683-s001.zip › microorganisms-3924203-supplementary.pdf]

Supplementary materials

Figure S1

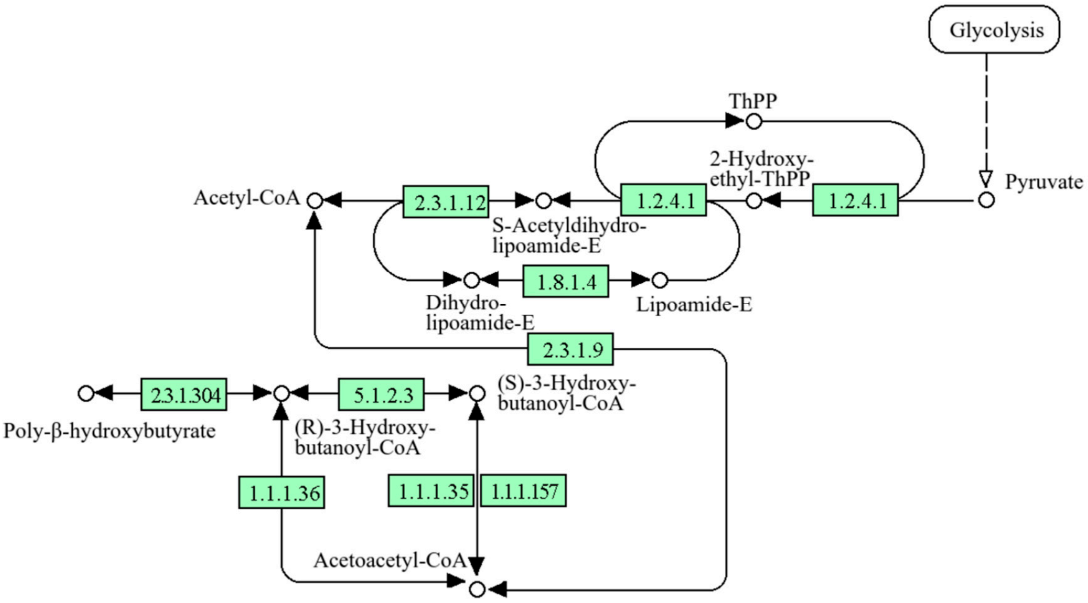

Figure S1. Metabolic pathway for PHA production obtained from KEGG mapper, green rectangles indicate that strain LNSP4103-1^{T} possesses the enzyme.

Figure S2

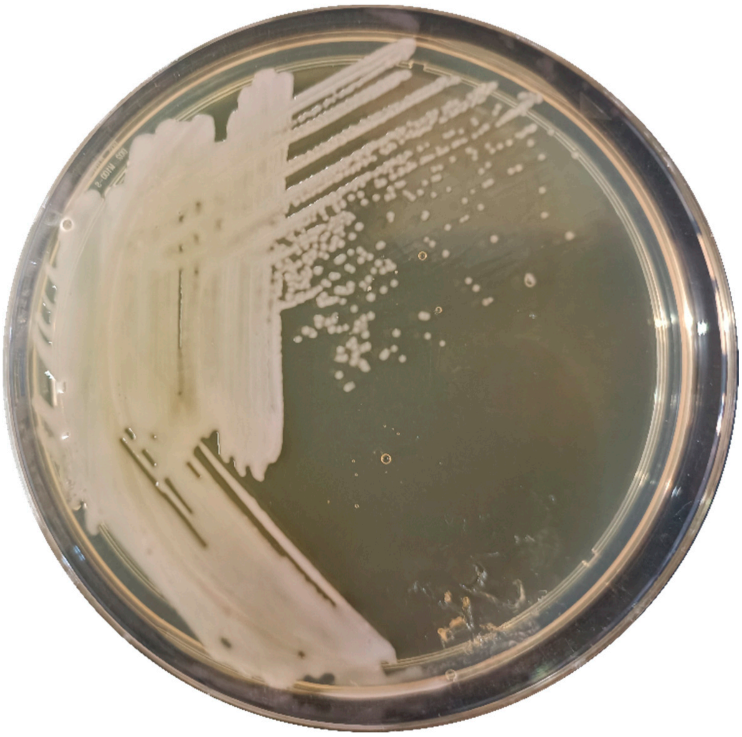

Figure S2. Colony morphology of the *Billgrantia hypersalina* LNSP4103-1^T in TSA after 72 h incubation at 30°C.

Figure S3

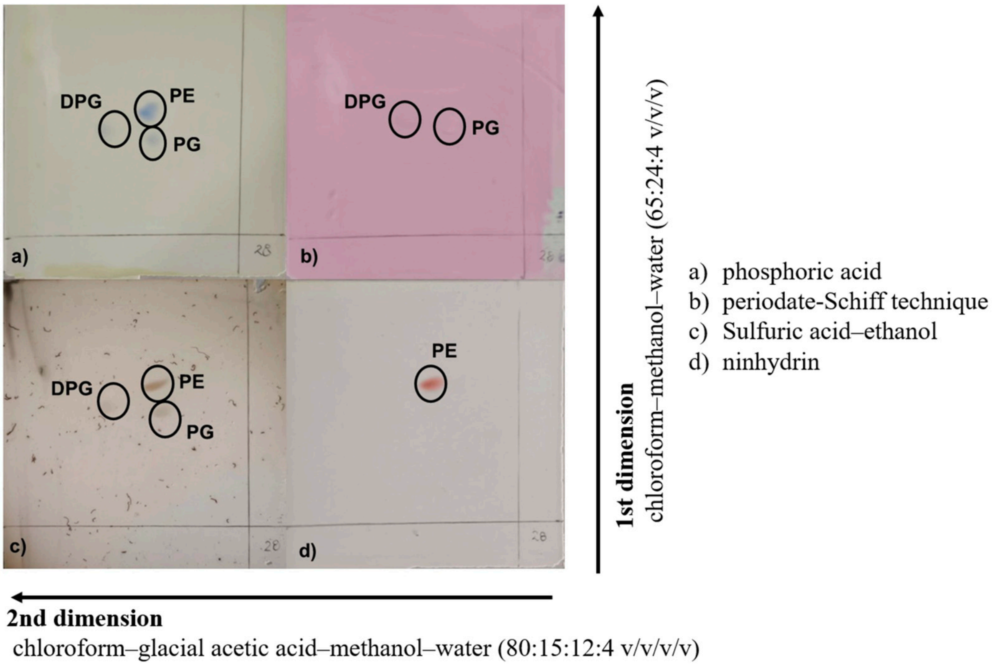

Figure S3. Polar lipid profile of *Billgrantia hypersalina* LNSP4103-1^{T}; major polar lipids detected were diphosphatidylglycerol (DPG), phosphatidylethanolamine (PE), and phosphatidylglycerol (PG).

Figure S4

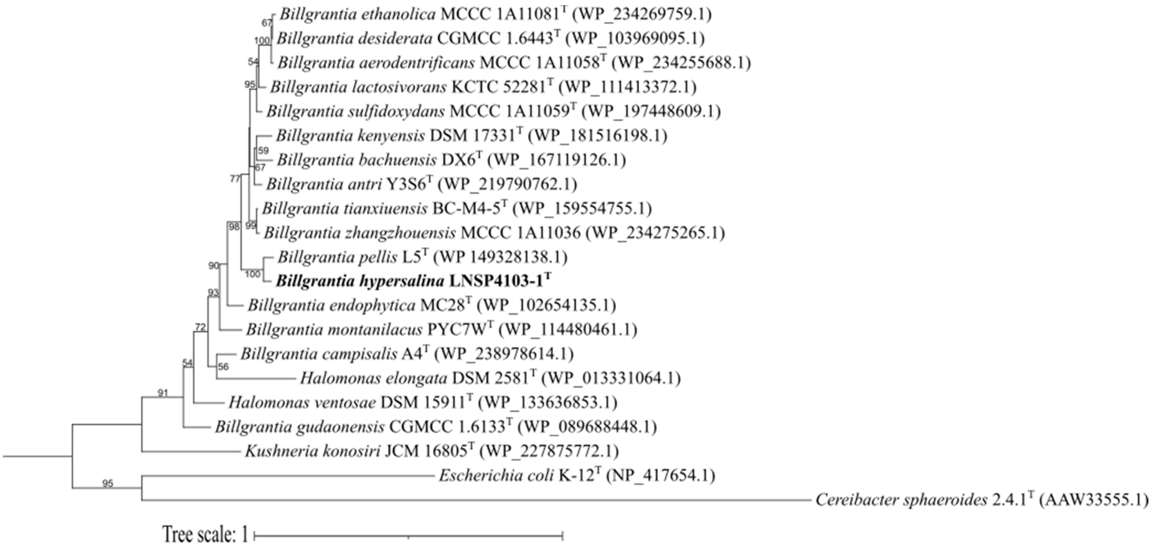

Figure S4. Phylogenetic reconstruction of the protein octaprenyl diphosphate synthase (ispB), the tree was constructed with the maximum likelihood method, using the LG + R3 (General Matrix) model. Nodes show bootstrap percentages ≥50% (1000 replicates). The tree scale was 1 substitution per amino acid, the protein decaprenyl diphosphate synthase (ispA) *Cereibacter sphaeroides* 2.4.1^{T} was used as outgroup.

Table S1. 16S rRNA gene similarity matrix for members of the genus *Billgrantia*.

	1	2	3	4	5	6	7	8	9	10	11	12	13	14	15	16	17	18	19	20	21	23
1. *B. diversa* D16761^{T}	100																					
2. *B. bachuensis* DX6^{T}	91.6	100																				
3. *B. antri* Y3S6^{T}	94.2	94.9	100																			
4. *B. pellis* L5^{T}	96.1	92.1	96.5	100																		
5. *B. azerbaijanica* TBZ202^{T}	93.2	92.5	94.8	95.7	100																	
6. *B. endophytica* MC28^{T}	89.8	96.1	93.7	90.9	91.4	100																
7. *B. montanilacus* PYC7W^{T}	94.1	91.1	93.7	96.1	93.7	91.2	100															
8. *B. lactosivorans* CFH 90008^{T}	95.4	92.9	96.6	97.3	95.9	91.3	94.8	100														
9. *B. saliphila* LCB169^{T}	94.4	94.2	97.9	96.7	94.1	92.8	94.2	95.9	100													
10. *B. desiderata* DQD230^{T}	90.4	96.5	94.7	92.3	91	94.1	89.8	93.2	93.9	100												
11. *B. gudaonensis* SL014B69^{T}	91.1	93.6	95.6	93.9	97	92.6	92.3	93.8	94.6	91.9	100											
12. *B. kenyensis* AIR2^{T}	87.7	93.9	91.7	89.7	88.8	92.1	87.7	89.9	91.2	95.2	90	100										
13. *B. campisalis* 4A^{T}	88.5	91.2	91.5	90.6	89.4	90.4	89.7	89.4	91.8	91.5	90.4	93.4	100									
14. *B. desiderata* FB2^{T}	95.2	93.8	95.5	96	95.5	92	94.3	97.9	94.6	93.2	94	89.8	89.2	100								
15. *B. hypersalina* LNSP4103-1^{T}	92.5	95.1	96.2	94.4	93.2	93.7	92	95.2	95.6	95.5	93.6	92.4	90.4	94.5	100							
16. *B. aerodenitrificans* CYD9^{T}	87.2	93.3	91.6	89.3	88	91.9	88.1	90.1	91.4	94.9	89	98	91.9	89.6	92.2	100						
17. *B. chromatireducens* AGD 83^{T}	91	92.9	94.9	92.7	92.9	93.3	96.3	93.6	94.1	91.9	94.3	89.9	90.2	93.4	93.4	90.6	100					
18. *B. ethanolica* CYT311^{T}	87.2	93.3	91.8	89.5	88.1	91.6	88	90.4	91.3	95.1	89.2	97.9	91.9	89.9	92.6	99.6	90.4	100				
19. *B. sulfidoxydans* CYN12^{T}	87.3	93.6	92	89.7	88.8	91.6	87	90.9	91.2	95.5	89.9	97.9	91.8	89.8	92.9	98.1	89.3	98.5	100			
20. *B. tianxiuensis* BCM45^{T}	87.6	93.6	91.9	89.7	88.8	91.7	87	90.8	91.4	95.4	89.8	98	92	90	92.8	98.3	89.3	98.7	99.6	100		
21. *B. zhangzhouensis* CXT311^{T}	87.2	93.4	91.7	89.3	88.6	91.6	87.1	90.4	91	95.1	89.7	97.8	91.7	89.6	92.4	97.9	89.5	98.2	99.3	99.4	100	
23. *Vreelandella aquamarina* DSM 30161^{T}	89.9	92.4	94.1	91.9	92.1	92.4	93.1	92.3	92.6	91.1	93.5	89.1	89.3	92.4	92.5	89.4	96	89.6	89.1	88.9	89	100

Table S2. Enzymes are involved in PHA metabolism.

EC number	Gene	Enzyme
1.2.4.1	*aceE*	Pyruvate dehydrogenase
1.8.1.4	*lpd*	Dihydrolipoyl dehydrogenase
2.3.1.12	*aceF*	Dihydrolipoxylysine acetyltransferase
2.3.1.9	*phaA*	Acetyl-CoA acetyltransferase
1.1.1.35	*fadB*	3-hydroxyacyl-CoA dehydrogenase
1.1.1.36	*phaB*	Acetoacetyl-CoA reductase
5.1.2.3	*fadJ*	3-hydroxybutyryl-CoA epimerase
2.3.1	*phaC*	Polyhydroxyalkanoate synthase subunit

Table S3. Genes involved in *"salt-out"* strategies or compatible solute production in strain LNSP4103-1^{T}.

Description	Gene	Enzyme
Glycine/Betaine	*betA*	Choline dehydrogenase
	betB	Betaine-aldehyde dehydrogenase
	betI	Transcriptional regulator of the family *bet*
	betL	Glycine/betaine transporter
	betT	Choline-glycine/betaine transporter
	betT_1	High affinity protein for choline transport
	betT_2	High affinity protein for choline transport
	betT_3	High affinity protein for choline transport
	gbsA	Betaine aldehyde dehydrogenase
	opuAA	Glycine/betaine transporter
	opuAB	Glycine/betaine transporter
	opuCA	Glycine/betaine/carnitine/choline transporter
	soxA	Thiosulfotransferase-S
	soxB	2'-hydroxybiphenyl-2-sulphinate desulphinase
	soxG	sarcosine oxidase gamma subunit
	yehW	Glycine/betaine permease uptake system
	yehY	Glycine/betaine permease uptake system
	yehZ	Glycine/betaine-binding protein
Ectoine	*asd*	Aspartate-semialdehyde dehydrogenase
	ectA	L-2,4-diaminobutyric acid acetyltransferase
	ectB	Diaminobutyrate-2-oxoglutarate transaminase
	ectC	L-ectoin synthase
	ectT	Ectoin/Hydroxyectoin transporter
	doeA	Ectoin hydrolase
	doeB	N2-acetyl-L-2, 4-diaminobutyrate deacetylase
	lysC	Aspartate kinase
Glutamate/Proline	*gltB*	Glutamate synthase (NADPH)
	gdhA	Glutamate dehydrogenase (NADP+)
	gudD	Glucarate dehydratase

	putA	1-pyrrolin-5-carboxylate dehydrogenase
	proA	Glutamate-5-semiladehyde dehydrogenase
	proB	Glutamate-5-kinase
	proC	Pyrroline-5-carboxylate reductase
	opuE	Proline osmoregulation transporter

Table S4. Genes involved in the "*salt-in*" strategies of strain LNSP4103-1ᵀ.

Description	Gene	Enzyme
Primary sodium pumps (Na^+)	*nqrA*	Na^+-subunit A of NADH-quinone reductase translocation
	nqrB	Na^+-subunit B of NADH-quinone reductase translocation
	nqrC	Na^+-subunit C of NADH-quinone reductase translocation
	nqrD	Na^+-subunit D of NADH-quinone reductase translocation
	nqrE	Na^+-subunit E of NADH-quinone reductase translocation
	nqrF	Na^+-subunit F of NADH- quinone reductase translocation reductase
	mrpA	Na^+/H^+ antiporter subunit A
	mrpB	Na^+/H^+ antiporter subunit B
	mrpC	Na^+/H^+ antiporter subunit C
	mrpD	Na^+/H^+ antiporter subunit D
	mrpE	Na^+/H^+ antiporter subunit E
	mrpF	Antiporter subunit F of Na^+/H^+
	mrpG	Anti-carrier subunit G of Na^+/H^+
	mnhE	Anti-carrier subunit E1 of Na^+/H^+
	nhaD	Anti-carrier of Na^+/H^+
	nhaP	Anti-carrier of Na^+/H^+
Potassium homeostasis	*trkA*	Potassium uptake protein
	trkH	Potassium uptake protein
	trkI	Potassium uptake protein
	fkpB	Peptidyl- prolyl cis-trans isomerase
	mscL	Large-duct mechanosensitive channel
	kefF	Glutathione-regulated potassium efflux pump
Osmoregulation	*glpD*	Glycerol-3-phosphate dehydrogenase aerobic
	glpE	Thiosulfate sulfurtransferase
	glpG	Rhomboid protease
	glpK	Glycerol kinase
	glpR	Glucagon receptor
